# Docetaxel-Loaded Pluronic P123 Polymeric Micelles: *in Vitro* and *in Vivo* Evaluation

**DOI:** 10.3390/ijms12031684

**Published:** 2011-03-04

**Authors:** Zhihong Liu, Donghua Liu, Lili Wang, Juan Zhang, Na Zhang

**Affiliations:** School of Pharmaceutical Science, Shandong University, 44 Wenhua Xi Road, Ji’nan 250012, Shandong, China

**Keywords:** docetaxel, Pluronic P123, micelles, cytotoxicity, anticancer efficacy

## Abstract

In this work, novel docetaxel (DTX) -loaded Tween 80-free Pluronic P123 (P123) micelles with improved therapeutic effect were developed. The freeze-dried DTX-loaded P123 micelles (DTX-micelles) were analyzed by HPLC, TEM and DLS to determine the DTX loading, micelle morphology, size, respectively. The *in vitro* cytotoxic activity of DTX-micelles in HepG2, A549 and malignant melanoma B16 cells were evaluated by MTT assay. The corresponding *in vivo* antitumor efficacy was assessed in Kunming mice bearing B16 tumor after intravenous administration. The DTX-loading and efficiency into the micelles were 2.12 ± 0.09% and 86.34 ± 3.32%, respectively. The DTX-micelles were spherical with a mean particle size of 50.7 nm and size distribution from 22 to 84 nm, which suggested that they should be able to selectively accumulate in solid tumors by means of EPR effect, with a zeta potential of −12.45 ± 3.24 mV. The *in vitro* release behavior of DTX from DTX-micelles followed the Weibull equation. Compared with Duopafei^®^, DTX-micelles showed higher cytotoxicity against HepG2 (P < 0.01), A549 (P < 0.05) and B16 (P < 0.01) cells. In addition, DTX-micelles exhibited remarkable antitumor activity and reduced toxicity on B16 tumor *in vivo*. The tumor inhibition rates (TIR) of DTX-micelles was 91.6% *versus* 76.3% of Duopafei^®^ (P < 0.01). These results suggested that P123 micelles might be considered as an effective DTX delivery system.

## 1. Introduction

Taxoids, paclitaxel (Taxol^®^) and docetaxel (Taxotere^®^), represent a novel class of antineoplastic drugs [1]. Docetaxel (DTX) is an inhibitor of microtubule depolymerization and has a broad antitumor activity against a variety of solid tumors [2], including breast [3–5], non-small cell lung cancer [6], ovarian [7] as well as gastric [8], head and neck [9], and prostate carcinomas [10].

However, its clinical efficacy is limited due to its poor solubility, low selective distribution, fast elimination *in vivo*, *etc.* Presently, Taxotere^®^ and Duopafei^®^ contain a high concentration of nonionic surfactant polysorbate 80 (Tween 80^®^) [11–13], which has been associated with several hypersensitivity reactions (e.g., fluid retention, neurotoxicity, musculoskeletal toxicity and neutropenia) [14,15]. In order to eliminate the Tween 80^®^-based vehicle and in an attempt to increase the drug solubility, lately, a number of alternative formulations have been developed, including liposomes [16–18], nanoparticles [19], microemulsions [20,21], drug conjugates [22–24]. The rationale behind these approaches is to increase antitumor efficacy of DTX while reducing systemic side effects.

In the past decade, polymeric micelles have been extensively studied for their prominent superiorities among the emerging nano-scopic carrier systems [25–27]. Polymeric micelles have a core-shell structure with diameters typically smaller than 100 nm. The hydrophobic core can serve as a microenvironment for incorporating hydrophobic drugs such as anticancer drugs by hydrophobic interaction. The hydrophilic outer shell serves as a stabilizing interface between the hydrophobic drug and the external medium, which can avoid the micelles being quickly taken up by the reticuloendothelial system (RES) after intravenous administration. It provides several advantages including drug solubilization, controlled drug release, escaping from RES uptake, and tumor targeting by enhanced permeability and retention (EPR) effect [28,29].

Several DTX-loaded polymeric micelles had been studied, including poly(ethylene oxide)-block-poly(butylene/styrene oxide)(PEO-b-P(SO/BO) micelles [30], poly(ethylene glycol)-block-poly(epsilon-caprolactone)(PEG-b-PCL) micelles [31], and monomethoxy-poly(ethylene glycol)-block-poly(L-lactide)/DTX (MPEG-b-PLLA/DTX) conjugates [24], which could notably solubilize and protect the anticancer drug docetaxel (DTX) from degradation. However, recent developments indicate that nanomaterials could not only serve as inert carriers, but also as biological response modifiers. One representative of such materials is Pluronic block copolymers that are amphiphilic synthetic polymers, composed of hydrophilic poly (ethylene oxide) (PEO) blocks and hydrophobic poly (propylene oxide) (PPO) blocks, arranged in triblock structure: PEO–PPO–PEO.

Several Pluronic block copolymers are listed in U.S. Pharmacopoeia and are approved for various medical uses as formulation excipients [32]. Pluronic micelles represent a novel type of nanomedicines that can increase solubility, improve circulation time, and release drugs at the target sites. Furthermore, Pluronic molecules display important biological activities of their own. Specifically, they can inhibit P-glycoprotein (P-gp), a drug efflux protein that hinders distribution of many drugs to the brain, intestine, and multidrug-resistant (MDR) tumors [11,33,34].

The aim of this study was to develop a new Tween 80-free, polymeric micellar formulation for DTX, intended to be intravenously administered. To achieve this purpose, Pluronic P123 (PEO_20_-PPO_65_-PEO_20_) with longer hydrophobic blocks, which was chosen for its commercial availability, biocompatibility and safety [35,36], was used to produce amphiphilic micelles for DTX. Paclitaxel (PTX)-loaded P123 micelles were studied by Han *et al.* [34]. The results showed P123 micelles may efficiently load, protect and retain PTX in the biological environment. Moreover, they could increase blood circulation time and reduce the distribution in the liver.

DTX-loaded P123 micelles were prepared by thin-film hydration method [11]. The micelles were characterized in terms of morphology, particle size and zeta potential. *In vitro* drug release was assessed using the dialysis bag diffusion technique. *In vitro* cytotoxic activity of DTX-micelles was performed using HepG2, A549 and malignant melanoma B16 cells. Finally, *in vivo* tumor growth inhibition of DTX-micelles was also investigated in Kunming mice bearing B16-tumor.

## 2. Results and Discussion

### 2.1. Preparation of DTX-Micelles

On the basis of optimization with single factors, orthogonal experiment design was employed for further optimization taking the entrapment efficiency as index. The variables in our studies were as follows: the weight of DTX, the weight of P123, the volume of oil-phase and water-phase.

The stability issue of polymeric micelles *in vitro* or *in vivo* has been an important challenge of micelle investigators [37]. Lyophilisation is a common procedure to increase the long-term stability of pharmaceutical formulations. In order to avoid disaggregation of DTX-micelles *in vitro*, the micelles solutions were freeze-dried to store. With 2% manicol as a protective excipient, a brittle and white color lyophilized powder was gained. The lyophilization process with manicol was successfully applied for lyophilization of DTX-micelles formulations. The DTX loading in the micelle formulation was stable enough for clinical application.

The optimized formulation was repeated in triplicate. The samples were analyzed before lyophilization and after reconstitution. In the initial formulation, the DTX-loading and efficiency into the micelles were 2.35 ± 0.08% and 92.07 ± 1.77%, respectively. After lyophilization and reconstitution, the DTX-loading and efficiency into the micelles were 2.12 ± 0.09% and 86.34 ± 3.32%, respectively. The solubility of DTX in micelles was increased up to about 0.8 mg/mL. This concentration is high enough to be used in clinical studies (<0.74 mg/mL).

### 2.2. Physicochemical Characterization of DTX-Micelles

**Figure 1.**
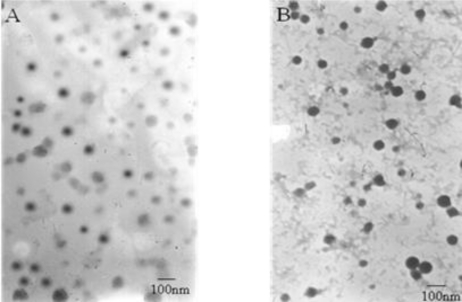
Transmission electron micrograms of DTX-micelles. (**A**) fresh-prepared micelles (×72,000); (**B**) freeze-dried micelles (×72,000).

**Figure 2.**
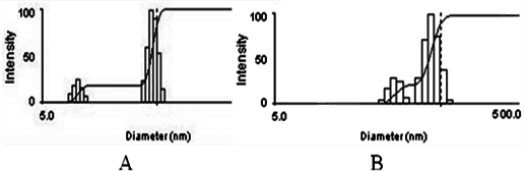
Size distribution of DTX-micelles determined by DLS. (**A**) fresh-prepared micelles; (**B**) freeze-dried micelles.

Figure 1A shows the TEM image of fresh-prepared DTX-micelles, which indicates that the self-assembled micelles are well dispersed as individual particles with spherical shape. Furthermore, DTX-micelles were found to have an average diameter of 38.9nm and size distribution from 9 to 55 nm (Figure 2A) and the zeta potential of −10.56 ± 2.34 mV. As shown in Figure 1B, freeze-dried DTX-micelles suspended in deionized water were still spherical with a mean particles size of 50.7 nm and size distribution from 22 to 84 nm (Figure 2B) and the zeta potential of −12.45 ± 3.24 mV.

The DLS analysis of reconstituted DTX-micelles showed only a small size increase after the lyophilization process, thus keeping the micellar carrier system. Moreover, the <100 nm diameter of polymeric micelles suggests that DTX-micelles should be able to selectively accumulate in solid tumors by means of EPR effect.

### 2.3. *In Vitro* Drug Release

**Figure 3.**
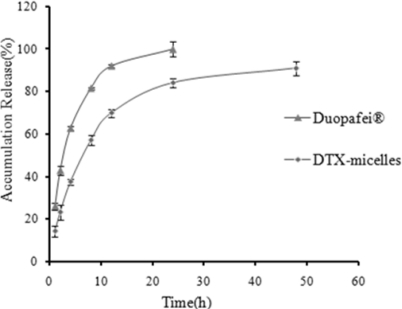
Accumulative DTX release from Duopafei^®^ and DTX-micelles in PBS (phosphate buffer solution, pH 7.4) containing 0.5% Tween 80 at 37 ± 0.5 °C (n = 3).

The *in vitro* release profile of DTX-micelles was investigated in PBS (phosphate buffer solution, pH 7.4) containing 0.5% Tween 80. The *in vitro* release behavior of DTX-micelles presented as the accumulative percentage release was shown in Figure 3. The release profiles of DTX-micelles were fitted with five different model equations, including zero-order kinetics, first-order kinetics, Higuchi equation, Weibull and Ritger-Peppas equations. The criterion for selecting the most appropriate model was based on best goodness-of-fit (R^2^ values). The results showed that the release of DTX from DTX-micelles followed the Weibull equation: lnln(1/(1−Q/100)) = 0.738lnt − 1.796 (r = 0.9935). It was obvious that DTX released much slower from DTX-micelles than from Duopafei^®^. The DTX-micelles released approximately 84.05% DTX during 24h, which was consistent with the release behavior of paclitaxel(PTX)-loaded P123 micelles [34]. In contrast, the release of DTX from Duopafei^®^ was faster and about 100% of the drug was released after being immersed for 24 h.

Normally, three basic mechanisms, namely swelling/erosion, diffusion and degradation are present for the release of the loaded drug from polymeric particles [38]. Any or all of these mechanisms may occur in a given release system. The hydrophilicity of the polymer would determine the uptake speed of water during the course of release. With the uptake of water, the micelle particles would swell and allow the drug within to diffuse through the pores. The difference between the release behavior of DTX from Duopafei^®^ and DTX-micelles may be attributed to the fact that the drug was encapsulated into the core of micelles [39]. The disintegration of the micelles after dilution was a relatively slow process. The drug released from micelles mainly through dissolution and diffusion.

### 2.4. *In Vitro* Cytotoxic Activity

The *in vitro* cytotoxic activity of Duopafei^®^, blank micelles and DTX-micelles was assessed by MTT assay in HepG2, A549 and B16 cells. The half maximal inhibitory concentration (IC50) values were listed in Table 1. The range of concentrations of DTX was from 0.01–20 μM. The drug concentration played a major role in the *in vitro* cytotoxicity of DTX. DTX-micelles and Duopafei^®^ showed similar concentration-dependent growth inhibition for all cell lines. However, each cell line exhibited different sensitivities to DTX-micelles. HepG2 cells were the most sensitive to DTX-micelles, with an IC50 value of 0.34 μM. DTX-micelles had decreased the IC50 values for all the cell lines with a statistical significance compared to Duopafei^®^, indicating that DTX-micelles showed higher cytotoxicity against these cells. This decrease in IC50 can result from an inhibition of cell growth or cell cytotoxicity [40].

Micelles were associated with the cells and internalized together with the entrapped drug in the cytoplasm, probably via endocytic mechanism. The improved interaction and intracellular localization led to the increased cytotoxicity comparable to that of Duopafei^®^ [11].

### 2.5. *In Vivo* Tumor Growth Inhibition Study

**Figure 4.**
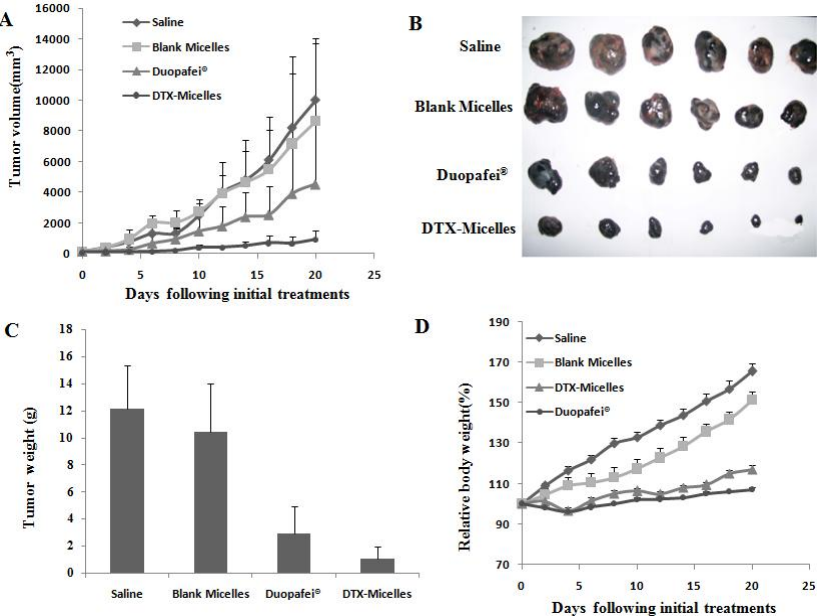
The *in vivo* antitumor effect of DTX-micelles. (**A**) the changes of tumor volumes of the tested groups; (**B**) the typical photographs of excised sarcomas from the tested groups; (**C**) the weights of excised tumor mass; (**D**) the variation of relative body weight of the mice with time. * P < 0.05, ** P < 0.01 *versus* Duopafei^®^.

The *in vivo* antitumor effect of DTX-micelles was assessed by intravenous administration using Kunming mice bearing B16 tumor as the model animals. The treatments were injected via the tail vein once a week for three weeks. Figure 4A shows changes of tumor volumes. It was found that the tumor volumes of DTX-micelles group were smaller than those of Duopafei^®^ group, indicating that DTX-micelles might more effectively inhibit tumor growth. It should be noted that the difference in tumor volumes among the groups of DTX-micelles and blank micelles as well as saline was highly significant (P < 0.01). Figure 4B shows typical photographs of excised sarcomas from the tested groups, which provide a direct visual representation of the tumor-suppression effect.

Table 2 lists the tumor inhibition rates of all the tested groups. The DTX-micelles group showed significant tumor inhibition rates (TIR = 91.6%). The weights of excised tumor mass were shown in Figure 4C (P < 0.05, DTX-micelles group *versus* Duopafei^®^ group). Figure 4D shows the variation of relative body weight of the mice with time. The results indicated that the mice experienced a slight weight loss of either DTX-micelles group or Duopafei^®^ group, while the extent of weight loss of DTX-micelles was much smaller than that induced by Duopafei^®^. The analysis of body weight variations can be used to define the systemic toxicity. These results lead to a conclusion that DTX-micelles generate less toxicitiy to normal organs than Duopafei^®^ when administered intravenously. Moreover, we also observed that the mice receiving Duopafei^®^ were in a weak state, in the aspects of movement and spirit, whereas no obvious alteration was observed in the micelles-treated animals.

In brief, it was shown that the antitumor efficacy of DTX-micelles was greatly superior to that of Duopafei^®^ in B16 tumor bearing mice model, which was consistent with the *in vitro* cytotoxicity test above. When encapsulated into micelles, DTX could reach the solid tumor site through EPR effect and maintain the effective therapeutic concentration for a longer period of time. The unique core-shell architecture of polymeric micelles with a diameter of several tens of nanometers might allow prolonged blood circulation and preferential accumulation in solid tumors [41–43].

The use of polymeric micelles as drug carrier may reduce the toxicity of the incorporated drug. In general, the toxicity of the whole formulation is investigated while results of the micelles itself are not described. So, there should be a specific emphasis on the toxicity of the “empty” non-drug loaded micelles. This is especially important when slowly or non-degradable micelles are used for drug delivery which may show persistence and accumulation on the site of the drug delivery, eventually resulting in chronic inflammatory reactions [44]. The results of an *in vitro* cytotoxicity test and *in vivo* tumor growth inhibition study showed that P123 micelles itself had a certain toxicity, which may be due to non-degradable micelles accumulated *in vivo*. The real reasons are unknown. Understanding clearance kinetics of P123 micelles would be important in understanding their potential for adverse effects. It is encouraging that DTX-micelles possess greater efficiency to solid tumors and less toxicity to normal organs than Duopafei^®^ in this work. It is worthy of further study for clinical application. The micelles formulations allowed stopping usage of Tween 80 which causes serious hypersensitivity reactions.

## 3. Materials and Methods

### 3.1. Materials

Pluronic P123 (P123) was purchased from Sigma (China). DTX and Duopafei^®^ were obtained from Qilu Pharmaceutical Co., Ltd (Jinan, China). Ultra-purified water was used throughout. All reagents for HPLC analysis, including acetonitrile and methanol were of HPLC grade. Other chemicals and reagents were of analytical grade, obtained commercially.

Human hepatocellular liver carcinoma (HepG2), lung adenocarcinoma (A549) and murine malignant melanoma (B16) cell line were obtained from Shandong Institute of Immunopharmacology and Immunotherapy (Shandong, China). 3-(4,5-dimethylthiazol-2-yl)-2,5-diphenyl tetrazolium bromide)(MTT) was purchased from Sigma-Aldrich (China).

### 3.2. Animals

Female Kunming mice (18–22 g) were supplied by Laboratory Animals Center of Shandong University, Jinan, China. The animals were used following the guidelines of the Ethical Committee for Animal Experiments of Shandong University. The animals were acclimatized at a temperature of 25 ± 2 °C and a relative humidity of 70 ± 5% under natural light/dark conditions for at least 24 h before dosing.

### 3.3. Preparation of DTX-Micelles

DTX-micelles were prepared by thin-film hydration method [11]. Briefly, 4 mg DTX and 150 mg P123 were dissolved in 3 mL acetonitrile in a round-bottom flask. The solvent was removed by rotary evaporation at 40 °C for about 30 minutes to obtain a solid DTX/copolymer matrix. Residual acetonitrile was removed under vacuum overnight at room temperature. Then, the resultant thin film was hydrated with 5 mL water at 40 °C, then stirred at 500 rpm for 1 h to obtain a clear micellar solution, which was filtered through 0.22 μm filters (Millipore) to remove the non-encapsulated drug, followed by lyophization.

### 3.4. Determination of Docetaxel Content in Micelles

The drug concentration was determined by RP-HPLC method (SPD-10Avp Shimadzu pump, LC-10Avp Shimadzu UV-vis detector). A 65:35 (v/v) degassed mixture of acetonitrile and water was used as the mobile phase. The reverse phase column was Venusil XBP C-18. The column temperature was maintained at room temperature. The flow rate was set at 1.0 mL/min and the samples were monitored at 230 nm [19]. Sample solution was injected at a volume of 20 μL. The HPLC was calibrated with standard solutions 5 to 50 μg/mL of DTX dissolved in acetonitrile (correlation coefficient r = 0.9998). Micelles were dissolved in acetonitrile and vortexed to get a clear solution. Drug-loading (DL%) and encapsulation efficacy (EE%) were calculated by the following equations [11]:

(1) $$\text{DL}\%=\frac{\text{weight}\;\text{of}\;\text{the}\;\text{drug}\;\text{in}\;\text{micelles}}{\text{weight}\;\text{of}\;\text{the}\;\text{feeding}\;\text{polymer}\;\text{and}\;\text{drug}}\times 100\%$$

(2) $$\text{EE}\%\frac{\text{weight}\;\text{of}\;\text{the}\;\text{drug}\;\text{in}\;\text{micelles}}{\text{weight}\;\text{of}\;\text{the}\;\text{feeding}\;\text{drug}}\times 100\%$$

### 3.5. Physicochemical Characterization of DTX-Micelles

Transmission electron microscope (TEM) was performed to evaluate the surface morphology of micelles after negative staining with phosphotungstic acid solution (2%, w/v)[45]. The mean particle size and size distribution of the micelles were determined by dynamic light scattering (DLS)(Zetasizer 3000SH, Malvern Instruments, UK). Zeta potential was measured by the Laser Doppler Anemometry (LDA) on ZetaPlus Zeta Potential Analyzer (Brookheaven Instruments Corporation). The lyophilized DTX-micelles were reconstituted in water, and were analyzed for size by DLS, for morphology by TEM, for drug loading by HPLC. All measurements were performed at 25 °C. Experimental values were calculated from the measurements performed at least in triplicate.

### 3.6. *In Vitro* Release Studies

*In vitro* release of DTX from the polymeric micelles were carried out in an aqueous release medium (PBS, pH 7.4) containing 0.5% Tween 80 (w/v) to enhance the solubility of DTX [20], using the dialysis bag diffusion technique. First, aliquots of DTX-micelles and Duopafei^®^ were placed into the pre-swelled dialysis bags with 8–14 kDa molecular weight cutoff, and were immersed in 15 mL of release medium in screw-capped tubes, which were placed in a horizontal shaker bath maintained at 37 ± 0.5 °C and shaken at 100 rpm. At fixed time intervals, the dialysis bags were taken out and re-placed into new containers filling with 15 mL fresh medium. The concentrations of DTX in the samples withdrawn from the incubation medium were analyzed by HPLC as described above. Sink condition was maintained throughout the release period. Data obtained in triplicate were analyzed graphically (the percent accumulative amount of DTX released from micelles *versus* time plotted).

### 3.7. *In Vitro* Cytotoxic Activity

The *in vitro* cytotoxic activity of DTX-micelles was tested in HepG2, A549 and B16 cells using the MTT assay [46]. Briefly, cells were seeded in 96-well plates at the density of 4000 viable cells per well and incubated 24 h to allow cell attachment. Cells were then treated with a series of doses of Duopafei^®^, blank micelles, or DTX-micelles, respectively, at 37 °C. After 96 h of incubation, 20 μL of MTT (5 mg/mL) was added to each well of the plate. After incubating for additional 4 h, MTT was aspirated off and 200 μL/well of DMSO was added to dissolve the formazan crystals. Absorbance was measured at 570 nm and 630 nm by a microplate reader (FL600, Bio-Tek Inc., Winooski, VT). Untreated cells were taken as control with 100% viability and cells without addition of MTT were used as blank to calibrate the spectrophotometer to zero absorbance [47]. The results were expressed as mean values ± standard deviation of 3 measurements.

### 3.8. *In Vivo* Tumor Growth Inhibition Study

Kunming mice implanted with B16 cells were used to qualify the relative efficacy of DTX-micelles through intravenous administration. The mice were raised under specific pathogen-free circumstances and all of the animal experiments were performed in full compliance with guidelines approved by the Animal Care Committee of Shandong University.

The mice were subcutaneously injected at the right axillary space with 0.1mL of cell suspension containing 5 × 10^4^ B16 cells. Treatments were started after 9–10 days of implantation. The mice with tumor volume of about 100 mm^3^ were selected and this day was designated as ‘Day 0’.

On Day 0, the mice were randomly divided into four groups (6 mice per group): group1: saline; group 2: blank micelles; group 3: Duopafei^®^ (DTX concentration of 20 mg/kg; diluted in saline); group 4: DTX-micelles (DTX concentration of 20mg/kg; diluted in saline). The treatments were injected via the tail vein once a week for three weeks. All mice were tagged, and tumors were measured every other day with calipers during the period of study. The tumor volume was calculated by the formula (W^2^×L)/2, where W is the tumor measurement at the widest point and L stands for the tumor dimension at the longest point. Each animal was weighed at the time of treatment, so that dosages could be adjusted to achieve the mg/kg amounts. Animals were weighed every other day throughout the experiments. The body weights of mice were monitored as an index of systemic toxicity [48,49].

At the end of the experiment, the animals were killed, and the tumor mass was harvested, weighed and photographed. The tumor inhibition ratio (TIR) was calculated according to the follow equation: TIR(%) = ((W_c_−W_t_)/W_c_) × 100%, wherein W_c_ and W_t_ represent the mean tumor weight of control group and treatment group, respectively.

### 3.9. Statistical Analysis

All results are expressed as mean ± standard deviation. Paired Students’s t-test or ANOVA analyses were performed to demonstrate statistical differences (P < 0.05).

## 4. Conclusions

The objective of this study was to design Tween 80-free micelles loaded with the poorly soluble anticancer drug DTX. DTX was well incorporated into P123 micelles with high drug-loading coefficient and encapsulation efficacy. The obtained micelles had a spherical shape with a hydrodynamic diameter of about 50 nm. Cytotoxicity test against HepG2, A549 and mouse B16 cells showed that DTX-micelles had better *in vitro* cytotoxicity than Duopafei^®^. Furthermore, the *in vivo* antitumor effect was investigated. It was found that DTX-micelles also exhibited superior *in vivo* antitumor effect when compared to the commercially available DTX injection. These results provide evidence for the clinical superiority of this micellar formulation, which has demonstrated a better therapeutic index than Duopafei^®^. Furthermore, this work has been completed as part of a patent application. In future studies, it is planned to use tumor models that are more sensitive to DTX, such as human breast cancer (MCF-7), in athymic nude mice. Taken together, P123 micelles have a promising future in clinical application.

## Figures and Tables

**Table 1.** The half maximal inhibitory concentration values on HepG2, A549 and B16 cells incubated with Duopafei^®^, DTX-micelles and Blank micelles at 96 h (n = 3).

**Treatment type**	**IC_50_ values (μM)**		
	**on HepG2 cells**	**on A549 cells**	**on B16 cells**
Duopafei^®^	0.96 ± 0.05	0.74 ± 0.02	0.72 ± 0.10
DTX-micelles	0.34 ± 0.02**	0.44 ± 0.05*	0.49 ± 0.08**
Blank micelles	12.84 ± 0.12	29.62 ± 1.02	13.79 ± 0.24

Note:

* P < 0.05,

** P < 0.01 *versus* Duopafei^®^.

**Table 2.** The *in vivo* antitumor effects in B16 bearing mice (n = 6).

**Formulation**	**Tumor weight x ± SD(g)**	**Tumor inhibition rate (%)**	**P^a^**
Saline	12.18 ± 3.20	N.A.	N.A.
Blank micelles	10.43 ± 3.59	14.4	N.A.
Duopafei^®^	2.89 ± 2.04	76.3	<0.01
DTX- micelles	1.02 ± 0.91	91.6	<0.01

a P value in the t-test denoting statistical significance.
